# Evaluation of Back Pain and Lead Apron Use Among Staff at a District General Hospital

**DOI:** 10.7759/cureus.18859

**Published:** 2021-10-18

**Authors:** Stefanie Andrew, Mohamed R Abdelmonem, Suraj Kohli, Harshad Dabke

**Affiliations:** 1 Trauma and Orthopaedics, Salisbury NHS Foundation Trust, Salisbury, GBR; 2 Trauma and Orthopaedics, University Hospitals, Plymouth NHS Trust, Plymouth, GBR

**Keywords:** radiology, occupational health, staff, lead apron, back pain

## Abstract

Objectives

To evaluate the prevalence of back pain among staff who regularly use lead aprons, correlating this to their use, and improve the knowledge and understanding of lead apron use among staff.

Methods

A questionnaire study was undertaken from November 2018 to February 2019 on staff in departments using lead aprons on a routine basis (n = 59) defined as the study group (SG), and staff who did not wear lead aprons (n = 62) defined as the control group (CG). Additionally, a separate questionnaire (n = 43) was distributed to lead apron users regarding education and knowledge, following which an education session was set up and the staff was re-evaluated.

Results

The prevalence of back pain was higher in the SG; 63% (SG) versus 32% (CG). The proportion of staff that felt lead aprons (SG) or work (CG) was the cause of this back pain was also higher in the SG than the CG: 83% versus 37%. A significant proportion of staff was unaware of the lead equivalence, material, and types of lead aprons available, after education this improved; 92% of staff now think more carefully when choosing a lead apron.

Discussion

Back pain is prevalent among staff using lead aprons and a lack of education regarding their use is evident. This could result in time off work and lead to unsafe practices around ionizing radiation. Education improved the knowledge and understanding of lead apron use. This could lead to increased comfort and less strain on the back, potentially lowering the prevalence of back pain.

## Introduction

Radiation exposure in hospitals is an invisible but potentially dangerous hazard. The key ways to reduce exposure include shielding (using protective equipment) and reducing exposure time [1]. A lead apron is a standard tool for protection against radiation exposure. Lead aprons have been shown to potentially reduce radiation exposure by up to 99% depending on their thickness and correct use [2]. The UK’s Health and Safety Executive (HSE) guidelines on personal protection equipment state that an employee should be able to carry the weight of a lead apron without injury [3]. However, various studies have highlighted issues regarding the prevalence of back pain due to lead apron use. It has been suggested that wearing a 15-pound lead apron can place pressures of up to 300 pounds per square inch on the intervertebral discs [4]. ‘Interventionalist’s disc disease’ has been identified as a confirmed entity by Ross AM et al. [5] who reported that in comparison to orthopedic surgeons and rheumatologists, cardiologists had a higher proportion of back pain leading to increased time off work. Their study also highlighted a higher incidence of cervical disc prolapse and degenerative disc disease across multiple levels in the spine. Therefore, there is significant concern that lead apron use can contribute to musculoskeletal (MSK) pathologies and low back pain. It is well known that low back pain is common, affecting up to half of the UK population, with significant economic and social ramifications [6].

The ideal lead apron would be weightless and novel devices have been suggested whereby the weight of the lead apron is borne by a mobile gantry [7]. Other suggestions have included the use of acrylic shields or robotics during procedures [8]. However, the practicality of these implements may be challenging, meaning that the lead apron is likely to be the principal manner of radiation protection for the foreseeable future. Standing occupations have also been associated with higher levels of lower back pain, which adds further weight to the problem [9]. New or worsening back pain due to lead apron use could lead to time off work and result in unsafe practice around ionizing radiation.

There are a variety of lead aprons available, the older versions are heavy in weight, but there are newer versions available made from lighter-weight lead-composite or lead-free materials. These have been reported as approximately 30% lighter than the older lead aprons with the same lead equivalence thereby offering the same level of protection [10,11]. There are multiple designs of lead aprons available, including wrap-around or two-piece garments. Improvements have been made to help distribute the load; the top and kilt design allows the weight to be distributed so that not all is taken in the shoulders and upper back [11]. There is considerable variability between hospital trusts, most having a variety of garments available, from the older lead designs to the newer lead-free aprons with different shapes and sizes. Notably, most staff would expect to have a radiation protection induction when starting at a new hospital trust but there seems to be little education for staff regarding the different types of lead aprons available and how to choose the most appropriate one. This could have an impact on the level of mechanical and musculoskeletal stress ensued.

Aim/Objectives

The primary objective of this study was to assess if low back pain was caused or exacerbated by lead apron use among staff who regularly used lead aprons compared to those who did not, and to assess if this was related to the duration of use.

The secondary objective of this study was to assess if there was a lack of education with regards to types of lead aprons and whether or not these issues could be addressed to improve and reduce symptoms in the longer term. This study also evaluated whether the use of lead aprons in Salisbury District Hospital was in accordance with the standards set by the HSE guidelines and the ionizing radiation regulations.

Standards/Guidelines/Evidence base

The HSE guidelines on personal protection equipment state that an employee should be able to carry the weight of a lead apron without injury [3].

The Ionising Radiations Regulations (IRR) 1999 part II highlights that the levels of exposure should be restricted and the appropriate equipment made available, correctly stored, and cared for [3].

## Materials and methods

This prospective study was undertaken from November 2018 to February 2019 and involved a questionnaire-based survey of staff in relevant departments who did and did not use lead aprons on a regular basis. “Regular basis” was defined as the use of lead aprons for two days or more every week. Questionnaires (Appendix 1) were distributed to all staff members in orthopedic operating theatres, radiology, and interventional cardiology who used lead aprons on a regular basis and formed the study group (SG). Questionnaires (Appendix 2) were distributed to staff members who did not use lead aprons regularly and formed the control group (CG) which included staff working in the wards, the ED, outpatient clinic, administration, and physiotherapy department.

Basic demographic data was collected such as job role, department, age, and gender. The SG questionnaire evaluated the length of time lead aprons were worn, the previous history of back pain (before wearing lead aprons), worsening/new back pain, correlation of back pain to lead apron use, days off work due to pain and apprehension/minimizing time worn. The CG questionnaire evaluated the prevalence of back pain, duration of back pain, treatment undertaken, correlation of back pain to work, days off work due to back pain.

Ionizing radiation and lead apron education

Additionally, a separate questionnaire (Appendix 3) was distributed to lead apron users (n = 43) regarding whether staff had had an ionizing radiation induction, what influenced their choice of the apron, knowledge of lead equivalence, knowledge of the different types of the lead aprons, thyroid shield use and storage. The condition, weight, and quantity of lead aprons were also evaluated and documented.

In August 2019, following data collection, an educational session was set up for theatre staff presenting the findings of the survey and educating staff regarding lead apron use and types of apron available. The local radiation protection advisor led this session which also provided information to staff regarding radiation protection policies and guidance. A questionnaire (Appendix 4) was then circulated to staff (n = 13) who had attended the educational session, covering knowledge of lead apron materials, what now influences their choice of the apron, knowledge of lead equivalence and the different types of the lead apron, thyroid shield use and whether they will now change their practice. The lead aprons available to staff were also assessed. This involved documenting the number of aprons, size, design (e.g., top/skirt, one-piece, wrap-around), lead equivalence, type (light duty/heavy duty), material (lead/lead-free), weight, quality, and storage.

## Results

Study group (SG)

**Figure 1:**
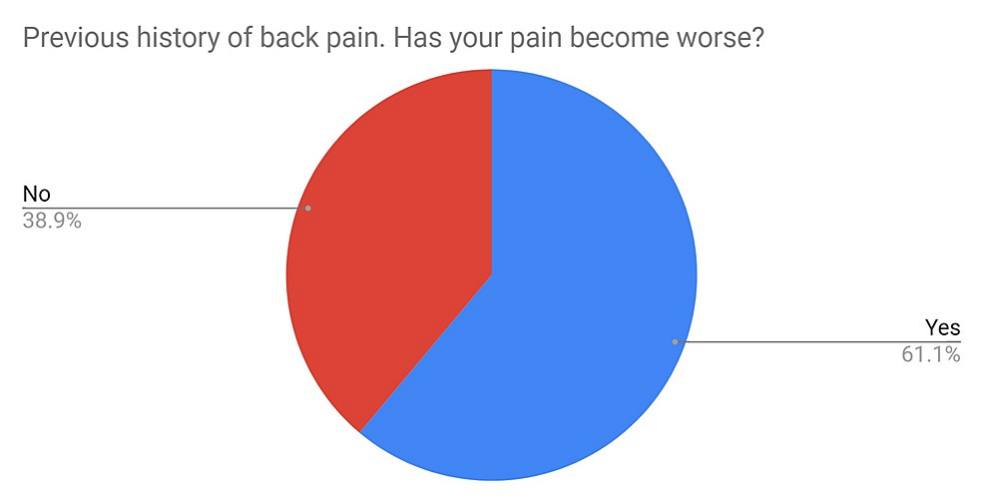
Pie chart depicting the percentage of staff with a previous history of back pain whose back pain is worse since wearing lead aprons.

This cohort consisted of 59 staff members with a male to female ratio of 32:26 and an average age of 39 years (range 22-64 years). A total of 30% of staff had a previous history of back pain, of these 61% felt that their back pain had worsened since wearing lead aprons (Figure 1). All bar one attributed their worsening back pain to the use of lead aprons.

**Figure 2:**
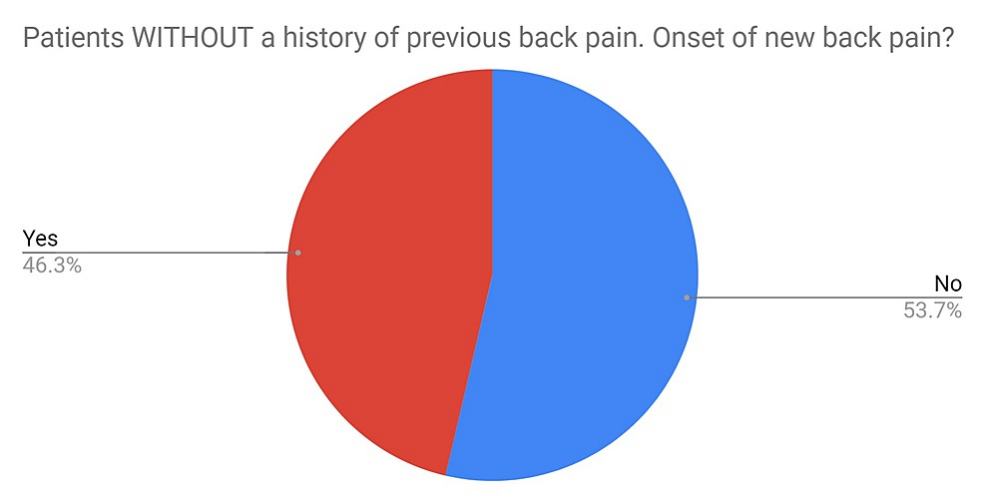
Pie chart depicting the percentage of staff with no previous history of back pain who developed new-onset back pain since wearing lead aprons.

A total of 46% of staff who did not have any previous history of back pain developed new back pain since wearing lead aprons (Figure 2).

**Figure 3:**
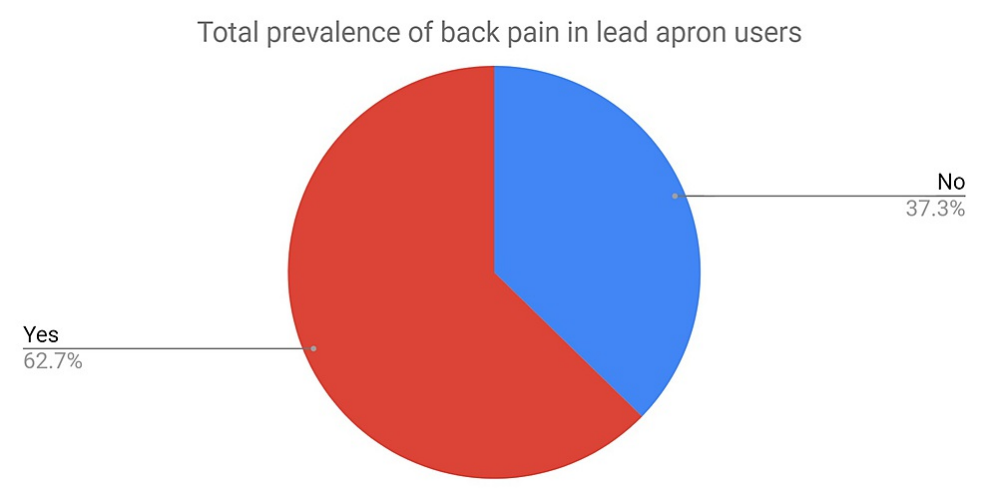
Pie chart depicting the total prevalence of back pain in the study group.

**Figure 4:**
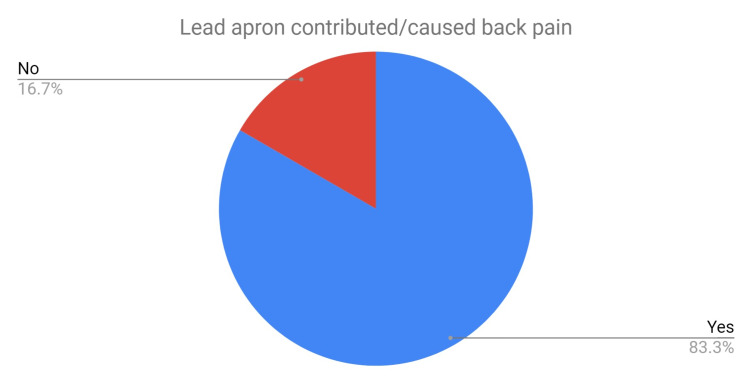
Pie chart depicting the percentage of staff who felt that their back pain was caused or contributed to by lead apron use.

The total prevalence of back pain in regular lead apron users was 62% (Figure 3) and of those, 83% felt that their back pain had been exacerbated or caused by wearing lead aprons (Figure 4). A total of 32% of staff in the SG were apprehensive about wearing lead aprons due to concerns of developing or exacerbating back pain and 56% had reduced the time that they used lead aprons. Three members of staff missed work due to pain from wearing lead aprons and three members of staff had even considered changing careers to avoid wearing them.

**Figure 5:**
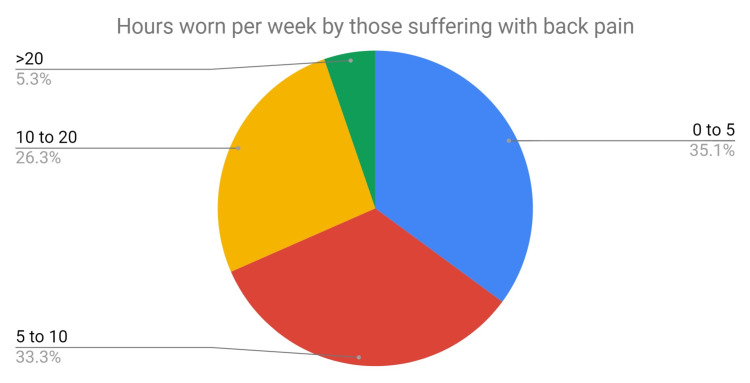
Pie chart depicting the number of hours that lead aprons were worn by those suffering from back pain.

Of the staff who were identified as suffering from back pain, the length of time lead aprons were worn was also evaluated (Figure 5). The prevalence of back pain was not associated with length of time worn, the results showed the following: 35% of staff with back pain wore lead aprons for an average of 0-5 hours per week, 33% with back pain wore them for 5-10 hours, and 26% with back pain wore them for 10-20 hours.

Control group (CG)

**Figure 6:**
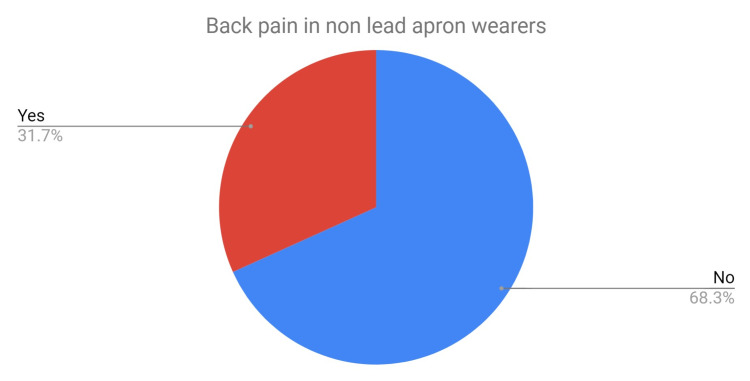
Pie chart depicting the percentage of back pain among staff who do not routinely use lead aprons.

There were 62 staff members with an average age of 41 years (range 21-65 years). A total of 32% suffered from back pain (Figure 6) and of these, 74% felt that their job contributed to this back pain, 37% felt that their job was the cause of their back pain. Four (21%) had to take time off work as a result of back pain caused by their job.

The two groups were similarly matched in age, however, there was an increased ratio of females to males in the CG. The prevalence of back pain was lower in the CG; 32% (CG) versus 63% (SG) (Table 1). The proportion of staff that felt work (CG) or lead aprons (SG) was the cause of this back pain was also lower in the CG than the SG: 37% versus 83%.

**Table 1:** Comparison between study and control groups.

	Study Group (SG)	Control Group (CG)
Total number	58	62
Average age (years)	39	41
M:F	32:26	7:56
No back pain	37%	68%
Total staff with back pain	63%	32%
Lead apron/Job caused back pain	83%	37%
Previous back pain	30%	32%
Worsening of previous back pain	61%	32%
Off sick due to back pain	3	4
Considering career change due to exacerbation of back pain	3	0

Ionizing radiation and lead apron education

**Figure 7:**
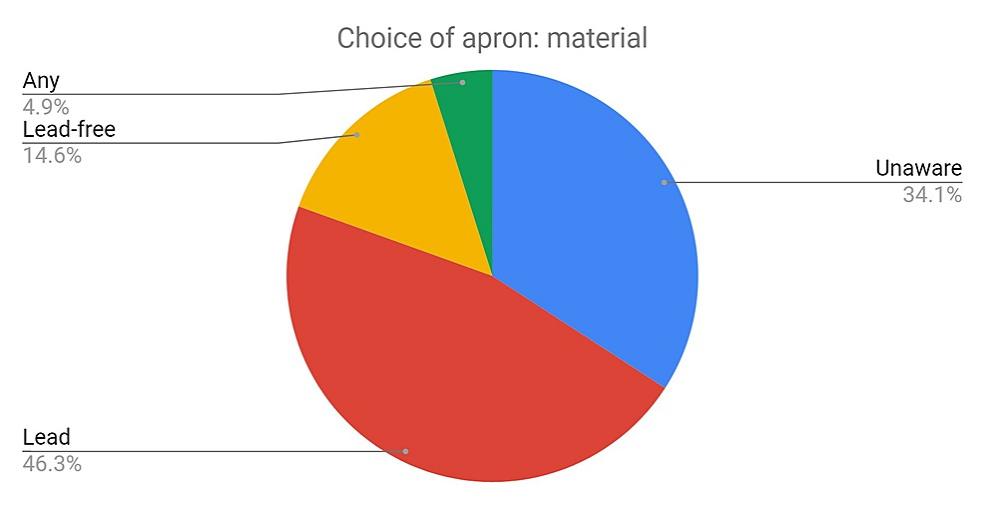
Pie chart depicting staff choices regarding lead apron material.

A total of 56% of staff surveyed had never had an induction covering ionizing radiation protection. A total of 56% of staff stated that weight was the most important factor that affected their choice of a lead apron. A total of 53% were unaware of the difference between the lead (Pb) equivalent protection of aprons. A total of 35% were unaware of the difference between lead versus lead-free aprons (Figure 7). A total of 65% did not wear thyroid shields, mostly because they were unavailable.

**Figure 8:**
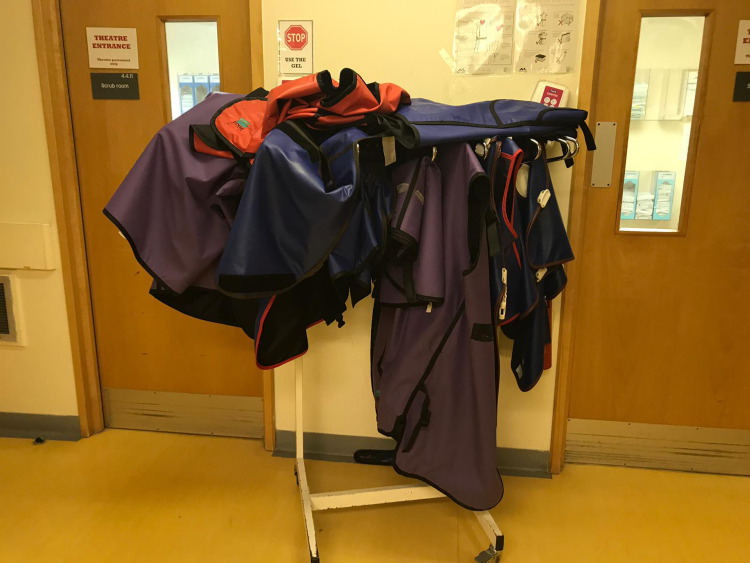
Photograph of lead apron storage outside of main theatres.

A total of 93% of staff allegedly always store their lead aprons correctly, however, our random inspections did not confirm that (Figure 8).

**Figure 9:**
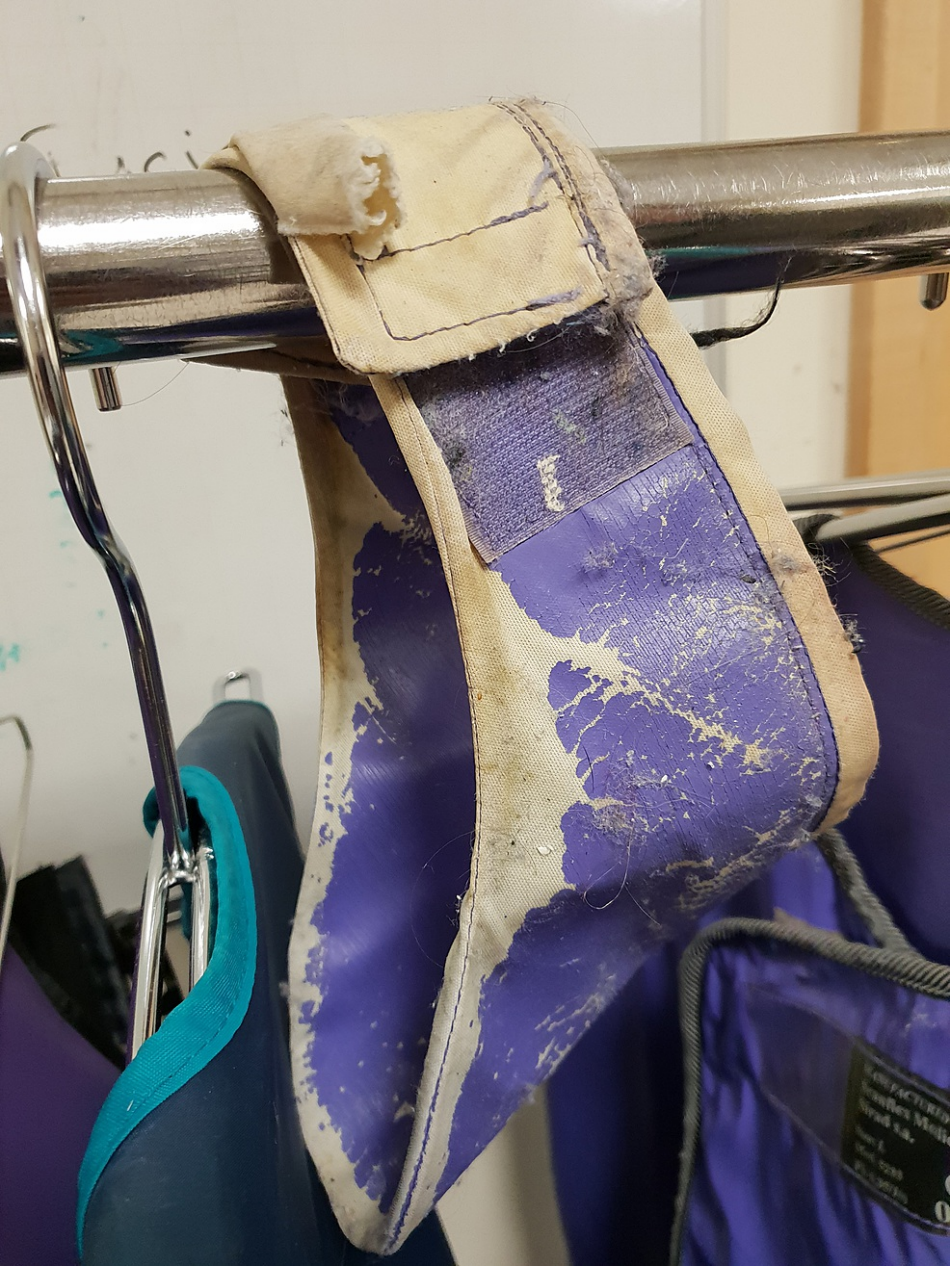
Photograph of the poor condition of thyroid shields available to staff.

Evaluation of lead apron weights revealed that the “light duty” lead aprons on average weighed 10% more than the heavy-duty lead-free aprons. Generally, there were large discrepancies between the number of tops versus skirts and the different sizes available. There were minimal large/extra-large aprons available. The quality of lead aprons and thyroid shields ranged from good to very poor (Figure 9).

**Figure 10:**
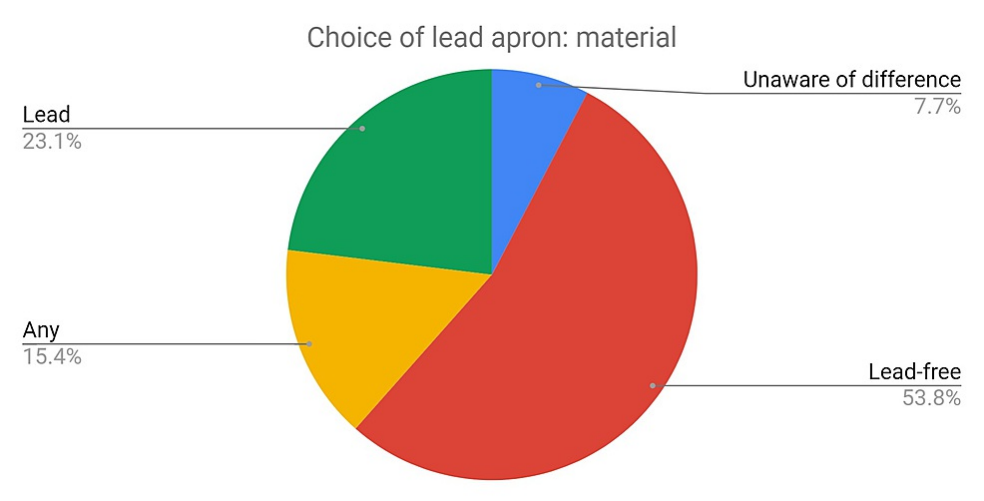
Pie chart depicting the staff choices regarding lead apron material.

Following the introduction of an educational session, data were collected in August 2019. After the session, 92% of the staff now think more carefully when choosing a lead apron. When making their choice, most are now aware of the differences and are making their choices accordingly; only 7.7% (previously 35%) were unaware of the difference between lead versus lead-free aprons (Figure 10). A total of 54% (previously 14.6%) chose lead-free aprons and 77% chose light-duty aprons.

**Figure 11:**
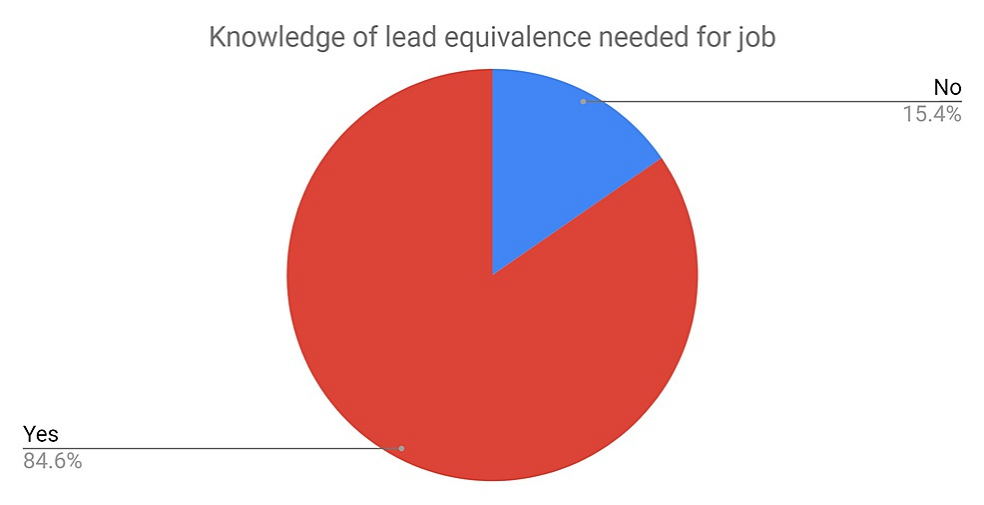
Pie chart depicting the percentage of staff who know the correct lead equivalence for the job following education.

Previously 53% were unaware of the difference between the Pb equivalent protection of the aprons, in contrast now 85% of staff know that the correct Pb equivalence for their job is 0.25 (Figure 11). The thyroid shield use was similar.

## Discussion

Low back pain has been reported to affect approximately one-third to one-half of the population in the UK [6]. Back pain in itself accounts for up to 40% of sickness absence in the UK [12] which has significant economic ramifications. It is complex and has many different treatment modalities [13]. This study has shown that back pain is more prevalent among staff who regularly used lead aprons (63%) compared to staff who do not (32%). The latter figure is in keeping with the point prevalence of low back pain which has been reported to be 33%, thereby confirming that regular use of lead aprons is associated with a disproportionately high prevalence of low back pain. These results are similar to previous studies which reported a high prevalence of back pain among lead apron users [4,5]. A high prevalence of spinal problems has been reported among interventional cardiologists, related to using lead aprons for long hours [14,15]. Our results confirm that operating theatre staff also experience more back pain than clinic or ward staff or the general population, highlighting a significant occupational hazard of the profession.

A total of 83% of staff in the SG who regularly used lead aprons, felt that their back pain had been exacerbated or caused by wearing lead aprons. Over one-third of these staff felt apprehensive about wearing lead aprons, three members of staff had missed work due to pain related to lead apron use, and were even considering changing careers to avoid wearing them. In comparison to these figures, only 37% of staff in the CG felt that their job, which did not require the use of lead aprons but involved standing for long periods, was the direct cause of their back pain. Although four staff members from the CG had taken sick leave due to back pain, none had considered changing careers.

The current study did not show that using lead aprons for long hours was necessarily associated with back pain with only 26% of staff in the SG experiencing back pain after using lead aprons for 10 hours and more. This finding is similar to that reported by Moore B et al., in 1992 who also found no correlation between the prevalence of back pain and duration of lead apron use [16].

The staff surveyed identified that the weight of the lead apron was the single most important factor in making a choice. However, a significant proportion of staff was unaware of lead equivalence or the difference in the material of lead aprons available. The majority of staff did not wear thyroid shields as these were unavailable and over half of the staff had not had an induction program in ionizing radiation. Almost all staff claimed to store their lead aprons correctly, but a snapshot of the department (Figure 8) revealed this not to be the case. Correct storage of lead aprons has been reported to be crucial in preventing cracks or holes from developing, which can reduce their integrity and hinder their protective ability [11].

This study highlighted that the introduction of an educational session produced a notable improvement in the knowledge and understanding of the safe usage of lead aprons. This is most likely because the session focused not only on radiation protection policies and guidance but also on lead apron use, types of apron available, lead versus lead-free options, sizing, fitting and safe level of lead equivalence. Imparting this information could enable staff to make an informed choice of the lead apron, which would suit their individual requirement. It seems logical that a well-fitting lead apron would be comfortable and could potentially reduce strain on the back which may help to decrease the high prevalence of back pain noted among regular users.

The quality and availability of lead protection were identified as being sub-standard, which can also impact staff comfort as they feel obliged to use ill-fitting gowns. This could be a conceivable cause for increased back pain.

Finally, there are some limitations to this study. Firstly, the two groups were not matched leading to potential confounding variables. The CG had a significantly higher proportion of females than males compared to the SG. It has been reported that the prevalence of chronic back pain is higher among females [6], however, despite the CG having a higher proportion of females, the overall prevalence of back pain was still lower than the SG. Secondly, only 13 staff members attended the educational session, a higher number of staff will need to be educated and evaluated to see if a change can be sustained and if this could influence the prevalence of back pain in the future. Lastly, this study is a snapshot of the current prevalence of back pain in a District General Hospital in the South of England which may not have the same demographics as other centers.

## Conclusions

These findings highlight that back pain is prevalent among staff using lead aprons regularly, and a lack of education is evident regarding their use. This can result in time off work and could lead to unsafe practices around ionizing radiation. Educational sessions have improved the knowledge and understanding of lead apron use, which could lead to a choice of better-fitting lead aprons with increased comfort and less strain on the back, potentially lowering the prevalence of back pain.

We propose the following recommendations for staff who wear lead aprons on a regular basis; all staff should have an induction and teaching on ionizing radiation. Moreover, all staff should be educated on the different types of lead aprons available and how to choose one appropriate to their line of work and body type. We also suggest that the lead apron stock in hospitals should be regularly audited and checked for any damage or defects. Further re-evaluations can take place to determine if these measures have improved staff comfort and back pain.

## Figures and Tables

**Figure 12:**
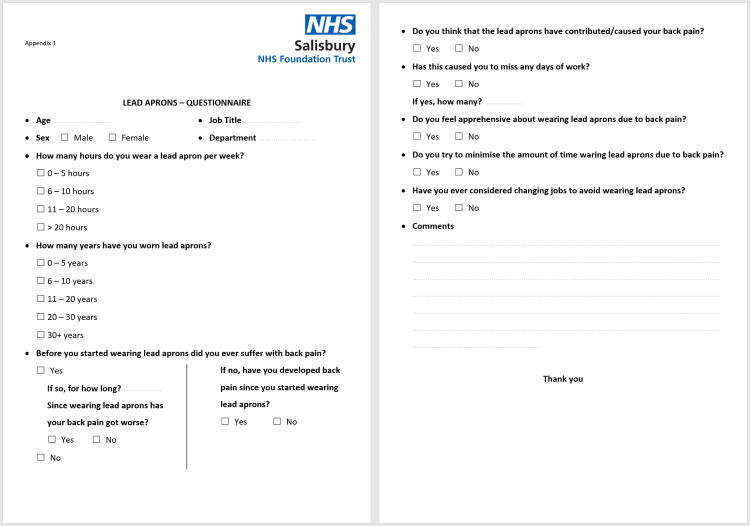
Questionnaire 1 (Appendix 1).

**Figure 13:**
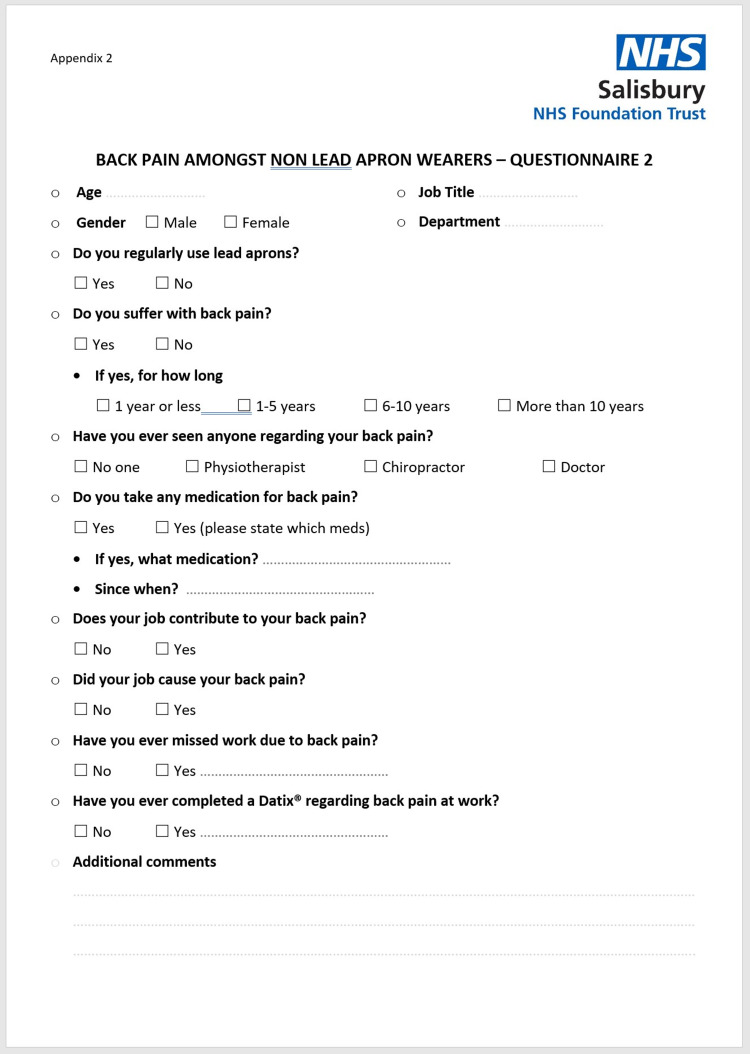
Questionnaire 2 (Appendix 2).

**Figure 14:**
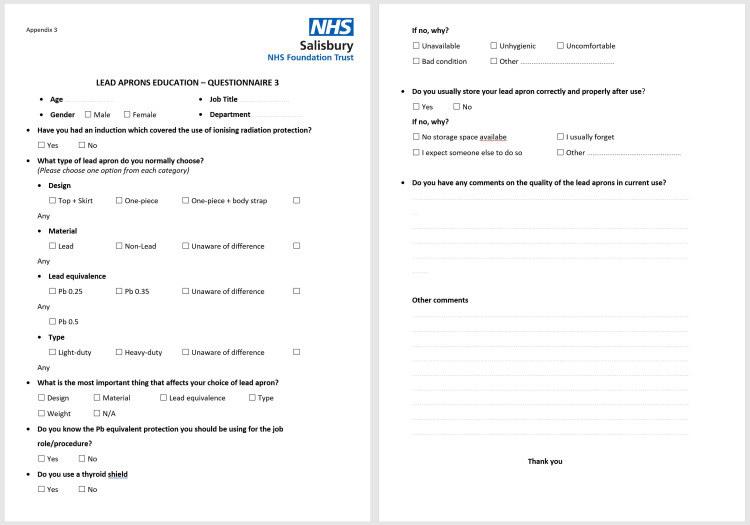
Questionnaire 3 (Appendix 3).

**Figure 15:**
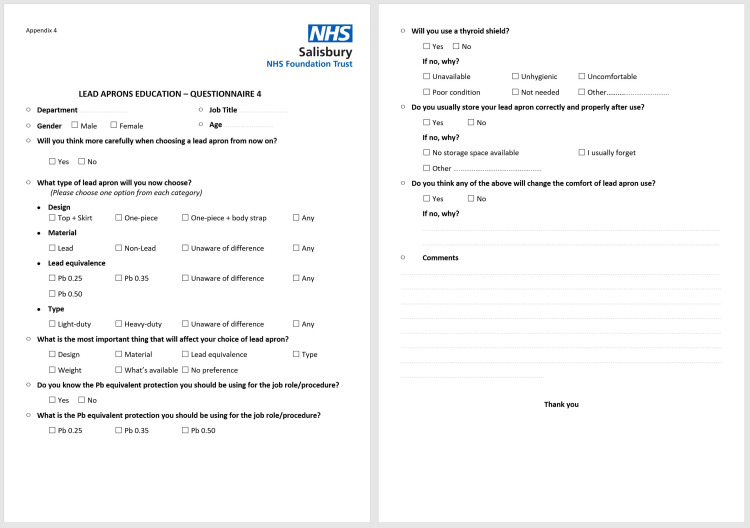
Questionnaire 4 (Appendix 4).

## References

[1] Singer G (2005). Occupational radiation exposure to the surgeon. J Am Acad Orthop Surg.

[2] Bushberg JT, Seibert JA, Leidholdt EM, Boone JM (1994). The Essential Physics of Medical Imaging. https://shop.lww.com/The-Essential-Physics-of-Medical-Imaging/p/9781975103224.

[3] (2017). Work with ionising radiation: Ionising Radiations Regulations 2017. Ionising radiations regulations.

[4] Khalil TM, Abdel-Moty EM, Rosomoff RS, Rosomoff HL (1993). Ergonomics in Back Pain: A Guide to Prevention and Rehabilitation. https://www.wiley.com/en-gb/Ergonomics+in+Back+Pain:+A+Guide+to+Prevention+and+Rehabilitation-p-9780471285441.

[5] Ross AM, Segal J, Borenstein D, Jenkins E, Cho S (1997). Prevalence of spinal disc disease among interventional cardiologists. Am J Cardiol.

[6] Fayaz A, Croft P, Langford RM, Donaldson LJ, Jones GT (2016). Prevalence of chronic pain in the UK: a systematic review and meta-analysis of population studies. BMJ Open.

[7] Pelz DM (2000). Low back pain, lead aprons, and the angiographer. AJNR Am J Neuroradiol.

[8] Rees CR, Duncan BW (2018). Get the lead off our backs!. Tech Vasc Interv Radiol.

[9] Tissot F, Messing K, Stock S (2009). Studying the relationship between low back pain and working postures among those who stand and those who sit most of the working day. Ergonomics.

[10] Cheon BK, Kim CL, Kim KR (2018). Radiation safety: a focus on lead aprons and thyroid shields in interventional pain management. Korean J Pain.

[11] Schueler BA (2010). Operator shielding: how and why. Tech Vasc Interv Radiol.

[12] (2020). The NHS Staff Council, Health, Safety and Wellbeing Partnership Group: Back in work: introduction and key messages. https://www.nhsemployers.org/sites/default/files/2021-08/Back%20in%20work%20part%201%20Introduction%20and%20key%20messages%20web%20final%2025%20March.pdf.

[13] (2020). National Institute for Health and Care Excellence: Low back pain and sciatica in over 16s: assessment and management. https://www.nice.org.uk/guidance/ng59/resources/low-back-pain-and-sciatica-in-over-16s-assessment-and-management-pdf-1837521693637.

[14] Klein LW, Tra Y, Garratt KN, Powell W, Lopez-Cruz G, Chambers C, Goldstein JA (2015). Occupational health hazards of interventional cardiologists in the current decade: results of the 2014 SCAI membership survey. Catheter Cardiovasc Interv.

[15] Goldstein JA, Balter S, Cowley M, Hodgson J, Klein LW (2004). Occupational hazards of interventional cardiologists: prevalence of orthopedic health problems in contemporary practice. Catheter Cardiovasc Interv.

[16] Moore B, vanSonnenberg E, Casola G, Novelline RA (1992). The relationship between back pain and lead apron use in radiologists. AJR Am J Roentgenol.

